# Diabetes and smoking are associated with dynapenic abdominal obesity in patients with chronic kidney disease: a cross-sectional study

**DOI:** 10.1590/1516-3180.2023.0232.R2.21102024

**Published:** 2025-05-26

**Authors:** Alessandra Fortes Almeida-Menezes, Maria Ester Pereira da Conceição-Machado, Maria Helena Lima Gusmão, Lílian Barbosa Ramos, Thais Vitorino Neves do Nascimento, Magali Teresópolis Reis Amaral, Jairza Maria Barreto-Medeiros

**Affiliations:** I Postgraduate Program in Food, Nutrition and Health, School of Nutrition, Universidade Federal da Bahia (UFBA), Salvador (BA), Brazil.; II Postgraduate Program in Food, Nutrition and Health, School of Nutrition, Universidade Federal da Bahia (UFBA), Salvador (BA), Brazil.; III Postgraduate Program in Food, Nutrition and Health, School of Nutrition, Universidade Federal da Bahia (UFBA), Salvador (BA), Brazil.; IV Postgraduate Program in Food, Nutrition and Health, School of Nutrition, Universidade Federal da Bahia (UFBA), Salvador (BA), Brazil.; V Postgraduate Program in Food, Nutrition and Health, School of Nutrition, Universidade Federal da Bahia (UFBA), Salvador (BA), Brazil.; VI Department of Statistics, Universidade Estadual de Feira de Santana (UEFS), Feira de Santana (BA), Brazil.; VII Postgraduate Program in Food, Nutrition and Health, School of Nutrition, Universidade Federal da Bahia (UFBA), Salvador (BA), Brazil.

**Keywords:** Renal insufficiency, chronic, Obesity, abdominal, Muscle strength, Waist circumference, Hand Strength, Nutritional status, Body mass index, Smoking, Diabetes

## Abstract

**BACKGROUND:**

No study has reported about the prevalence and factors associated with dynapenic abdominal obesity in patients with pre-dialysis chronic kidney disease (CKD).

**OBJECTIVE:**

Evaluation of the prevalence of dynapenic abdominal obesity and its relationship with sociodemographic, lifestyle, clinical, and nutritional variables in patients with CKD not dependent on dialysis.

**DESIGN AND SETTING:**

A cross-sectional study was conducted at the Nutrition and Nephropathy Outpatient Clinic (public service) in Bahia, Brazil.

**METHODS:**

This cross-sectional study was conducted on 102 patients of both sexes, aged ≥ 20 years. Dynapenic abdominal obesity (DAO) was defined as the simultaneous presence of dynapenia (handgrip strength less than the first tertile of the sample itself, according to sex and age) and increased waist circumference. Differences between groups with and without DAO were assessed using the Student’s Mann–Whitney t-test, Pearson’s chi-square test, or Fisher’s exact test. Associations were tested using bivariate and multivariate models with Poisson regression to calculate the prevalence ratio and 95% confidence intervals (PR; 95% CI).

**RESULTS:**

The mean age of the patients was 58.7 (standard deviation = 11.69); 50.5% were male, 51.6% were elderly, 41.8% had diabetes, 5.5% were smokers, 58.2% were abdominally obese, and 38.5% were dynapenic. DAO was identified in 18.7% of participants and was associated with diabetes mellitus (PR = 2.8; 95% CI = 1.12-6.99) and smoking (PR = 3.22; 95% CI = 1.16-8.96).

**CONCLUSION:**

Non-dialysis dependent patients with CKD showed a significant prevalence of DAO associated with smoking and diabetes mellitus.

## INTRODUCTION

The global prevalence of chronic kidney disease (CKD) is 13.4% higher among women than men.^1,2^ This percentage is expected to rise in the coming decades worldwide due to increased longevity^3^ and comorbidities, which contribute to diseases such as type 2 diabetes mellitus (DM) and hypertension.^4^ Therefore, it is essential to comprehend the changes that can occur as the disease progresses, allowing prevention and proper intervention.^5^ Among these changes, obesity is related to worsening muscle strength and kidney functions^6^, particularly excessive abdominal fat.

The condition in which dynapenia, defined as low muscle strength, and abdominal obesity (AO) are present simultaneously is characterized as dynapenic abdominal obesity (DAO),^7^ which may be present in patients with CKD due to inflammation, oxidative stress, metabolic acidosis, and insulin resistance.^8^ DAO is considered more severe for health than the isolated occurrence of AO or low muscle strength. This condition can increase the risk of loss of functional capacity, falls, hospitalization, and morbidity and mortality.^9-12^

In patients with chronic diseases, DAO should be better studied in terms of prevalence and factors associated with it, especially in patients with pre-dialysis CKD. In hemodialysis patients, the prevalence of DAO is approximately 20%^5^ and is associated with high morbidity, especially cardiovascular disease (CVD) and increased mortality.^13^ Therefore, the early detection of DAO and identification of its associated factors are essential for managing CKD and avoiding undesirable clinical outcomes. Factors such as smoking, which can increase the inflammatory response triggered by cigarette use,^14^ and the presence of DM should be investigated in patients with CKD, as they trigger inflammation, oxidative stress, and insulin resistance.^15^ In addition to these factors, physical inactivity, inadequate energy, protein intake, and other factors that can worsen clinical conditions in CKD are crucial.

## OBJECTIVE

This study aimed to assess the prevalence of DAO and its relationship with sociodemographic, lifestyle, clinical, and nutritional variables in patients with non-dialysis dependent CKD.

## METHODS

This cross-sectional study was conducted at the Nutrition and Nephropathies Outpatient Clinic of Professor Francisco de Magalhães Neto, a member of Professor Edgard Santos University Hospital Complex, Universidade Federal da Bahia (UFBA), Salvador, Brazil. The study was approved on September 24, 2012, by the Research Ethics Committee of Professor Edgard Santos Hospital Complex of the Federal University of Bahia (HUPES-UFBA) (protocol number 104,761/12). Signed informed consent forms were obtained from all patients before data collection.

The study included (convenience sample) clinically stable outpatients with CKD, with a glomerular filtration rate (GFR) between 89 and 15 ml/min/1.73 m^2^, aged > 20 years of both sexes.

Patients with hospital admission in the month before the beginning of the study, limb amputation, active neoplasia, immunological diseases, dialysis or kidney transplantation history, use of immunosuppressive drugs, acute kidney injury, severe liver disease, and CKD on dialysis were not included in the current study.

### DAO

DAO was defined as the coexistence of AO and dynapenia.^16^

AO was defined according to the waist circumference (WC) measured using an inelastic tape. This measurement was performed with the patient standing with the arms relaxed along the body. Thus, the tape was positioned at the midpoint between the iliac crest and the edge of the last rib, with the region free of clothing and the abdomen relaxed at the end of normal expiration.^17^ Measurements were taken in duplicate by the same evaluator, and the average between them was considered. The World Health Organization (WHO) does not differentiate WC by age group. Nevertheless, due to the lack of cutoff values for older adults, the WHO recommendation using the high-risk point (≥ 102 cm in men and ≥ 88 cm in women) was considered. Furthermore, for adults, the AO consideration was ≥ 94 cm for men and ≥ 80 cm for women.^18^

Dynapenia was identified by measuring handgrip strength (HGS) using a dynamometer (Bulb Dynamometer^®^).^19-21^ Patients were instructed to squeeze the dynamometer with maximum force in response to voice commands. The measurement was repeated three times, and the highest value was used for the study, according to the technique of Hillman et al.^22^ Dynapenia was considered when the HGS value was lower than the first tertile, according to sex and age.^12,23,24^ For adults, the following were considered low strength: females HGS < 11 kg, males HGS < 15 kg; in the elderly: females HGS < 10 kg and males HGS < 12.5 kg.

### Factors associated with DAO

Sociodemographic, lifestyle, clinical, and nutritional data were evaluated. Among the sociodemographic factors, sex, age, and educational level were evaluated. Education was categorized using the years of school attendance as 0–8, ≥ 9 years.^10^ Lifestyle-related factors were physical activity, alcohol consumption, and smoking. Physical exercise was classified into two categories: none or regular, with regular exercise considered for those who performed it at least three times a week for a minimum of 30 min every day.^25^ Alcohol consumption was assessed by asking patients whether they drank alcohol or not. Smoking habits were assessed by self-reporting, and based on the responses, the participants were categorized as never smokers or smokers.

For clinical factors, the presence of DM, duration of CKD, estimated glomerular filtration rate, use of statins, hemoglobin value, serum urea, and serum creatinine were evaluated. All biochemical tests were performed according to routine follow-up by a nephrologist, and the hospital laboratory adopted reference values. Serum urea was measured using an enzymatic method, and serum creatinine was measured using a modified Jaffé method (1960).

The diagnosis of DM was defined according to the criteria established by the American Diabetes Association,^26^ with fasting blood glucose ≥ 126 mg/dL, hemoglobin A1c ≥ 6.5%, random plasma glucose ≥ 200 mg/dL or use of antidiabetic drugs or based on the history of DM previously diagnosed by the doctor. Data on the time since the diagnosis of CKD were collected from the medical records of the patients. GFR was calculated and estimated based on serum creatinine from the proposed equation of the CKD Epidemiology Collaboration (CKD-EPI),^27^ and the CKD stage was classified using the Kidney Disease Improving Global Outcomes criteria.^28^

The nutritional factors assessed included body mass index (BMI), which was calculated by dividing weight by the square of height and continuously using the data. Body composition was evaluated using electrical bioimpedance measurements. Biochemical tests (serum dosage of total proteins and albumin-bromocresol green method) and food consumption (macronutrients and calcium and phosphorus consumption) were obtained from the collection of food records for 3 d (two weekdays and one weekend day) using Diet Pro version 5.0 (AS Sistemas, Viçosa, Minas Gerais, Brazil).

From the tetrapolar electrical bioimpedance (BIA Biodynamics^®^ P450 Bioimpedance Analyzer, Seattle, Washington, United States), the percentage of body fat and appendicular skeletal muscle mass (ASMM) were analyzed, as recommended by the (European Consensus on Definition and Diagnosis of Sarcopenia) (EWSOP2).^18,29^ The ASMM was obtained using the following equation: ASMM (Kg) = -3.964 + [0.227* (height^2^/resistance)] + (0.095* weight) + (1.384* sex) + (0.064* reactance), where height is in cm, sex information for male = 1 and female = 0, and resistance in ohms. Both were used as continuous variables.

### Statistical analysis

Data were presented as mean and standard deviation (SD) or median and interquartile range for continuous variables and as frequency and prevalence for categorical variables. Data normality was tested using the Shapiro–Wilk test. The difference between the two groups (DAO and Non-DAO - NDAO) was determined using Student’s t-test or Mann–Whitney U test, according to the normality of the variable. Categorical variables were compared using Pearson’s chi-square or Fisher’s exact tests.

The association between DAO and its factors was verified using Poisson regression with robust variance by calculating the prevalence ratio (PR) and 95% confidence interval (95% CI).

First, bivariate regression models were performed, and the variables that presented a P value ≤ 0.20 were considered in the multivariate model. Only variables with a P value < 0.05 remained in the final model. All the statistical analyses were performed using SPSS version 20.0 (IBM, Armonk, New York, United States).

## RESULTS

Of the 102 patients, 11 were excluded because they did not complete all protocol steps, such as HGS, anthropometry, and biochemical tests—the final sample comprised 91 patients.

The 91 patients who participated in this study had a mean age of 58.7 years (SD = 11.69); 49.5% were female, 51.6% were elderly, 75.8% were sedentary, 5.5% were smokers, 41.8% had DM, 84.9% had stages 3 and 4 CKD, and 41.8% were taking statins (Table 1).

**Table 1:** Sociodemographic, lifestyle, and clinical variables of patients with non-dialysis-dependent chronic kidney disease according to dynapenic abdominal obesity, 2013

Variables	TOTAL 91 (100%)	Dynapenic Abdominal Obesity		P value
		Present 17 (18,7%)	Absent 74 (81,3%)	
Women (n (%))	5 (49.5)	12 (70.6)	33 (44.6)	0.053^1^
Age (mean (SD))	58.7 ±11.69	57.18 ±12.23	59.09 ±11.61	0.504^3^
Elderly (≥ 60 years) (n (%))	7 (51.6)	08 (47.1)	39 (52.7)	0.675^1^
Educational level (0 to 9 years old) (n (%))	61 (67.0)	10 (58.8)	51 (68.9)	0.425^1^
Sedentary lifestyle (n (%))	69 75.8)	14 (82.4)	55 (74.3)	0.754^2^
Alcoholism (n (%))	18 (19.8)	03 (17.6)	15 (20.3)	1.000^2^
Smoking (n (%))	05 (05.5)	03 (17.6)	02 (02.7)	**0.043** ^2^
Diabetes (n (%))	38 (41,8)	12 (70.6)	26 (35.1)	**0.008** 1
CKD time (months)	36 (12-96)	15 (7-43)	38.5 (12-120)	**0.033** ^4^
GFR (ml/min)	37.1 (23.2-52.4)	36.8 (23.8-44.4)	37.1 (23.1- 54.9*)	0.885^4^
CKD stages 3 and 4 (n (%))	73 (84.9)	15 (88.2)	58 (84.1)	1.000^2^
Statin use (n (%))	38 (41.8)	10 (58.8)	28 (37.8)	0.114 ^1^
Urea (mg/dl)	67 (50-101.2)	78.5 (47.7-125.7)	64.0 (50-101.2)	0.665^4^
Creatinine (mg/dl)	1,7 (1.1-2.6)	1.7 (1.3-2.8)	1.7 (1.1-2.6)	0.887^4^
Hemoglobin (g/dl)	12.0 ±1.87	11.63 ±1.89	12.16 ±1.86	0.290^3^

^1^ Chi-square test;

^2^ Fisher’s exact test;

^3^Student’s t test;

^4^Mann–Whitney test.

SD = standard deviation; CKD = chronic kidney disease; GFR = glomerular filtration rate.

**Figure 1:**
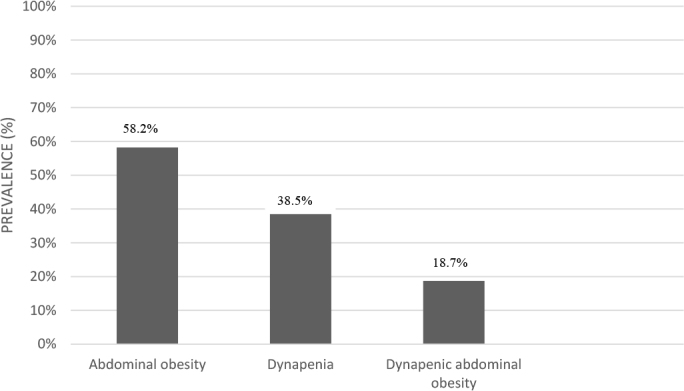
Prevalence of abdominal obesity, dynapenia, and dynapenic abdominal obesity in patients with non-dialysis-dependent chronic kidney disease, 2013.

**Figure 2:**
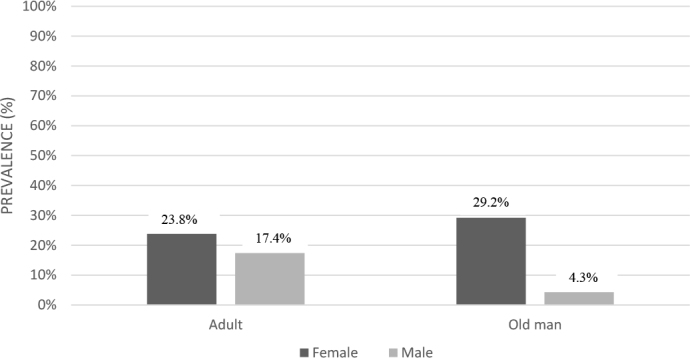
Prevalence of dynapenic abdominal obesity, according to sex and age, in patients with non-dialysis chronic kidney disease 2013.

AO was present in 58.2% (n = 53) of the patients, dynapenia in 38.5% (n = 35), and DAO in 18.7% (n = 17) (Figure 1). Elderly females had a higher prevalence of DAO (Figure 2). Among patients without DAO, 24.3% (n = 18) had dynapenia, and 48.6% (n = 36) had AO. The mean HGS was 10.20 kg (SD = 1.86) in the DAO group and 13.34 kg (SD = 2.65) in the non-DAO group.

In the DAO group, the frequency of diabetic participants (P = 0.008) and those with a smoking habit (P = 0.043) was significantly higher than that in the group without DAO. The mean time since CKD diagnosis was significantly shorter (P = 0.033) in participants with DAO than those without DAO (Table 1). Regarding the nutritional variables shown in Table 2, the most significant presence of adipose tissue was observed in the DAO group (P = 0.021). There were no significant differences in the other variables between the groups.

**Table 2:** Nutritional variables of patients with non-dialysis-dependent chronic kidney disease according to dynapenic abdominal obesity, 2013

Variables	TOTAL 91 (100%)	Dynapenic Abdominal Obesity		P value
		Present 17 (18,7%)	Absent 74 (81,3%)	
BMI (kg/m^2^)	25.16 (21.34-29.3)	27.18 (24.6-30.7)	24.7 (21.17-28.68)	0.054 ^2^
ASMM (Kg)	18.27 (15.06-21.67)	17.23 (15.62-20.10)	18.29 (15.0-22.4)	0.831 ^2^
Adipose tissue	16.7 (12.7-21.6)	20.6 (15.8-27.8)	15.3 (12.7-20.6)	**0.021** ^2^
Total proteins (g/dL)	7.7 ±0.64	7.72 ±0.76	7.68 ±0.62	0.822 ^1^
Albumin (g/dL)	4.3 (4.1-4.6)	4.3 (3.7-4.4)	4.3 (4.1-4.6)	0.465 ^2^
Daily energy intake (Kcal/day)	1664 (1315.5-2065.3)	1672.3 (1210-2190)	1650.3 (1315-2009)	0.968 ^2^
Daily energy intake (Kcal/Kg)	25.0 (19.9-34.2)	25 (16.5-33.8)	25 (20.1-34.3)	0.481 ^2^
Daily protein intake (g/day)	71.36 (60.26-101.5)	84.4 (59.0-101.3)	70.6 (60.7-101.5)	0.708 ^2^
Daily protein intake (g/kg)	1.2 (0.9-1.6)	1.1 (0.7-1.7)	1.2 (0.9-1.6)	0.628 ^2^
Carbohydrates (g/day)	238.9 (182.1-301.8)	217.0 (173-306.2)	242.1 (183.9-301.8)	0.768 ^2^
Carbohydrates (%)	57.9 ±7.94	57.4 ±8.07	57.99 ±7.96	0.815 ^1^
Lipids (g)	35.6 (30.0-50.1)	35.5 (32.8-48.1)	35.6 (28.8-50.1)	0.786 ^2^
Lipids (%)	19.9 (17.1-25.2)	19.5 (15.8-26.2)	19.9 (17.2-24.5)	0.768 ^2^
Calcium consumption (mg)	584.9 (402.3-744.9)	733 (408.8-801.8)	572 (402.3-734.3)	0.280 ^2^
Phosphorus consumption (mg)	1085.5 (838.6-1456.2)	1099.6 (839.5-1238.3)	1041.0 (838.6-1473.3)	0.977 ^2^

^1^ Mean and standard deviation, Student’s t-test;

^2^Median and interquartile range, Mann–Whitney test.

BMI = body mass index; ASMM = appendicular skeletal muscle mass.

Bivariate and multivariate analyses of the association between the variables evaluated and the DAO are presented in Table 3. In the multivariate analysis, adjusted for age, sex, and duration of CKD, DAO was associated with DM (PR = 2.80 95% CI = 1.12-6.99) and smoking (PR = 3.22; 95% CI= 1.16-8.96).

**Table 3:** Prevalence ratio between dynapenic abdominal obesity and demographic, lifestyle, clinical, and nutritional variables, 2013

Variables	_Bivariate_ PR (95% CI)	_Multivariate_ PR (95% CI)*
**Age group**		-
Adult	1.00	
Elderly	0.83 (0.35 -1.96)	
**Sex**		-
Male	1.00	
Female	2.45 (0.94 -6.40)	
**Smoking**		
No	1.00	1.00
Yes	3.69 (1.56 -8.72)	3.22 (1.16-8.96)
**Statin use**		-
No	1.00	
Yes	1.99 (0.83- 4.76)	
**Presence of Diabetes Mellitus**		
No	1.00	1.00
Yes	3.35 (1.30 – 8.71)	2.80 (1.12 -6.99)
CKD time	0.99 (0.98;1.00)	-
Body mass index	1.09 (1.01;1.17)	-
Adipose tissue	1.08 (1.03;1.14)	-

*Regression models are adjusted for age, sex, and CKD duration.

PR = prevalence ratio; CI = confidence interval; CKD = chronic kidney disease.

## DISCUSSION

The main findings of our study showed that the prevalence of DAO among patients with non-dialysis-dependent CKD is important. The prevalence was 3.2 times higher in smokers and 2.8 times higher in patients with DM.

In the present study, dynapenia showed high prevalence along with AO. A study by Alexandre et al.^9^ identified that dynapenia was evident in patients with increased WC.^9^ Muscle function may constitute a better prognostic factor for predicting mortality when compared to muscle mass in patients with AO.^30^ For this reason, due to its unfavorable results, investigating dynapenia is gaining importance in the academic community.^9^ Furthermore, a previous study reported that obesity is an independent risk factor for low muscle strength.^31^ Besides, AO has a more significant negative impact on metabolic abnormalities than general obesity.^32^

To the best of our knowledge, no previous study has investigated DAO in patients with non-dialysis-dependent CKD. In a cohort that followed older adults with DAO without CKD for 10 years, worsening of disability and mortality was observed compared to participants with low muscle strength or excess abdominal fat alone.^16^ In the literature research, we did not find any study on the prevalence of DAO in non-dialysis dependent CKD patients to compare with the results of our study. However, some studies have evaluated the prevalence of DAO during dialysis.

In a study by Tabibi et al.,^33^ who evaluated patients on peritoneal dialysis, a prevalence of 11.4% for dynapenic obesity and 31.6% for dynapenia was reported. These prevalence rates were lower than those reported in the present study in patients with non-dialysis dependent CKD. It is worth noting that in the above study, obesity was assessed by BMI, not by AO, as in the present study. Furthermore, dynapenia was assessed considering the cutoff points proposed by the Asian Working Group for Sarcopenia (AWGS).^34^ A study by Corrêa et al.,^5^ in older adults undergoing hemodialysis, identified a prevalence of 15.8% for AO, 25.9% for dynapenia, and 19.8% for DAO. These authors also evaluated AO by considering the WC cutoff points proposed by the WHO.

Considering the physiological changes during aging, such as the redistribution of body fat, the same cut-off points proposed for adults in elderly patients may be inappropriate. Therefore, this study defined AO based on WC categorized according to two different cutoff points.^18^

Dynapenia was assessed based on HGS tertiles. In a Brazilian study with older adults undergoing hemodialysis in the maintenance phase, the lowest tertile of male and female patients was considered dynapenic when HGS was ≤ 18 kg.^5^ Therefore, the HGS tertile was lower in the elderly in the present study (female HGS < 10 kg and male HGS < 12.5 kg). Contrastingly, we found a slightly higher prevalence of DAO; however, we used the reference of the population for dynapenia with values previously recommended for WC.

In our study, there were no significant differences in the prevalence of DAO between males and females or between adults and older people. Although a higher prevalence of dynapenic obesity is expected among older adults, it has been shown that this condition can develop in younger adults with catabolic diseases, such as CKD.^35^ This is clinically relevant because in patients with CKD, factors such as inflammation, oxidative stress, metabolic acidosis, decreased testosterone secretion, insulin resistance, growth hormone resistance, inadequate energy and protein intake, vitamin D deficiency, physical inactivity, increased protein catabolism, and dynapenia occur regardless of age.^8^

Currently, muscle strength (MS) is the most reliable measure of muscle function. The MS moderately correlates with strength in other body segments, so measuring grip strength can identify low muscle strength.^19^ A EWSOP2 report recommends values to consider the reduced handgrip strength (for men below 27 kg and women below 16 kg). However, in the current study, we chose to consider low strength and handgrip strength values lower than the first tertile of the sample itself, according to age and sex, because the cutoff points proposed by the EWSOP2 are not specific to the Brazilian population and CKD. Notably, other authors defined dynapenia as the lowest tertile of the sample itself.^12,13,23,24^

The time of CKD diagnosis was significantly shorter in the DAO group. This can be explained by the shorter duration of nutritional follow-up in the outpatient clinic. It has been observed that patients with CKD for a long time are under nutritional monitoring for a longer time, which positively affects their nutritional status.

Toxic chemicals present in cigarettes may explain the high prevalence of DAO in smokers. Smoking can increase inflammatory marker levels, reduce oxygen supply, and impair mitochondrial function. Moreover, chronic cigarette use appears to induce loss of muscle mass and dynapenia.^36,37^

However, the mechanisms underlying the harmful effects of obesity on muscle function have not been elucidated. Despite this, the accumulation of muscle fat and some metabolic abnormalities, such as increased oxidative stress, insulin resistance, and inflammation, have been identified as determinants,^38^ and oxidative stress induced by cigarette substances aggravate DAO.

It is also believed that, in this group, the longer time since DM diagnosis or chronic hyperglycemia may have contributed to the increased prevalence of DM. Accumulation of advanced glycation end products (AGEs), which cause diabetic vascular complications through oxidative stress and chronic inflammation, appears to contribute to reduced muscle strength in patients with DM.^39,40^

This effect on dynapenia in hyperglycemia induced by DM in patients with central obesity further facilitates muscle atrophy and loss of strength. This situation occurs because of the increase in the accelerated formation of AGEs, accentuated oxidative stress, and change to type I muscle fibers, which are less potent than type II.^38^

Both dynapenia and AO can cause metabolic changes. Therefore, it is necessary to hypothesize that people with both conditions would present an even more altered metabolic profile than those with only one condition.^41^ Furthermore, as muscle strength decreases faster than muscle mass, DAO occurs earlier than sarcopenic obesity (SO), so early detection of DAO in the early stages can prevent SO.^8^

Thus, screening for dynapenia and AO in clinical practice would be helpful in healthcare settings, easy to perform, and inexpensive. This will contribute to the development of clinical intervention policies to prevent loss of muscle strength and AO in patients with CKD who are not undergoing dialysis. Moreover, it improves the morbidity, mortality, and quality of life of these patients.

The strengths of this study include the assessment of a population with stable CKD that was not dependent on dialysis, the use of standardized techniques, and a team of trained nutritionists for data collection. Moreover, due to the absence of specific cutoff points for the study population, WC and HGS were evaluated considering values from the sample itself according to sex and age. However, our study had some limitations, such as a small sample size, which may have contributed to the absence of other associations with the DAO.

## CONCLUSION

The prevalence of DAO in non-dialysis dependent patients was essential and associated with smoking and DM. The assessment of DAO should be investigated in nutritional monitoring and future research, especially in patients with non-dialysis dependent CKD.

Identifying the combination of central fat distribution and reduced muscle strength in patients with CKD may help delay future clinical complications in this population and improve quality of life. In these patients, individuals associated with DM and smoking were more likely to develop this combination. Therefore, suitable glycemic control and smoking cessation may be beneficial in preventing and avoiding the progression of DAO in these patients.
